# Development of a statistical model for cervical cancer cell death with irreversible electroporation in vitro

**DOI:** 10.1371/journal.pone.0195561

**Published:** 2018-04-25

**Authors:** Yongji Yang, Michael A. J. Moser, Edwin Zhang, Wenjun Zhang, Bing Zhang

**Affiliations:** 1 Tumor Ablation Group, Complex and Intelligent Systems Research Center, East China University of Science and Technology, Shanghai, China; 2 Department of Surgery, University of Saskatchewan, Saskatoon, Saskatchewan, Canada; 3 Division of Vascular & Interventional Radiology, Department of Medical Imaging, University of Toronto, Toronto, Ontario, Canada; 4 Biomedical Science and Technology Research Center, School of Mechatronic Engineering and Automation, Shanghai University, Shanghai, China; University of South Alabama Mitchell Cancer Institute, UNITED STATES

## Abstract

**Purpose:**

The aim of this study was to develop a statistical model for cell death by irreversible electroporation (IRE) and to show that the statistic model is more accurate than the electric field threshold model in the literature using cervical cancer cells in vitro.

**Methods:**

HeLa cell line was cultured and treated with different IRE protocols in order to obtain data for modeling the statistical relationship between the cell death and pulse-setting parameters. In total, 340 in vitro experiments were performed with a commercial IRE pulse system, including a pulse generator and an electric cuvette. Trypan blue staining technique was used to evaluate cell death after 4 hours of incubation following IRE treatment. Peleg-Fermi model was used in the study to build the statistical relationship using the cell viability data obtained from the in vitro experiments. A finite element model of IRE for the electric field distribution was also built. Comparison of ablation zones between the statistical model and electric threshold model (drawn from the finite element model) was used to show the accuracy of the proposed statistical model in the description of the ablation zone and its applicability in different pulse-setting parameters.

**Results:**

The statistical models describing the relationships between HeLa cell death and pulse length and the number of pulses, respectively, were built. The values of the curve fitting parameters were obtained using the Peleg-Fermi model for the treatment of cervical cancer with IRE. The difference in the ablation zone between the statistical model and the electric threshold model was also illustrated to show the accuracy of the proposed statistical model in the representation of ablation zone in IRE.

**Conclusions:**

This study concluded that: (1) the proposed statistical model accurately described the ablation zone of IRE with cervical cancer cells, and was more accurate compared with the electric field model; (2) the proposed statistical model was able to estimate the value of electric field threshold for the computer simulation of IRE in the treatment of cervical cancer; and (3) the proposed statistical model was able to express the change in ablation zone with the change in pulse-setting parameters.

## Introduction

Electroporation is defined as the creation of micro/nanopores in the cell membrane by transmembrane voltages leading to an increase in cell membrane permeability. Originally, electroporation was used to treat tumors by creating reversible pores in the cell membranes of cancer cells, through which chemotherapeutic drug or plasmid DNA is delivered into intracellular structures to kill tumor cells—a process called electrochemotherapy (ECT) [1]. Based on ECT, Davalos *et al*. proposed the idea of using irreversible pores in the cell membrane to kill tumor cells [2] (without chemotherapy), which has received much attention in the pre-clinical and clinical studies as a monotherapy for cancer treatment [3–7]. Electroporation that generates unrecoverable pores in the cell membrane is termed irreversible electroporation (IRE), differentiating it from ECT. Specifically, irreversible pores are generated by increasing the transmembrane voltage to a critical threshold using high magnitude electric pulses (hundreds to thousands of V/cm) [2]. Unlike the case of ECT, the cell death that occurs in the process of IRE is due to the permanent membrane lysis and/or loss of homeostasis after the generation of irreversible pores in the cell membrane.

Although IRE was only introduced about a decade ago, many pre-clinical and clinical studies have shown that IRE has great potential for the ablation of different types of tumors. Compared with thermal ablation (e.g., radiofrequency ablation, microwave ablation, and laser ablation), IRE has two unique advantages in the therapy of tumors: (1) no collateral thermal injury, and (2) no heat-sink effect when the ablation occurs near large arteries or veins. The second advantage makes IRE appealing for the treatment of tumors which are located in critical positions (e.g. near a large size of blood vessel or a critical organ), at which the thermal ablation methods are unfavorable [7].

However there are some shortcomings with IRE, for example: (1) tumors larger than 3 cm in diameter are not generally amenable to treatment with IRE, and (2) the heterogeneous ablation in the target treatment zone, which can lead to the tumor recurrence. Our present study aims to address these shortcomings. The general idea was to develop a more accurate mathematical model with which the IRE process can be simulated. This idea is viable, as supported by the literature, e.g., the study to make an optimal pre-clinic IRE treatment planning with a mathematical model [8, 9], optimal design of IRE electrodes [10, 11], and understanding of the synergistic effectiveness of IRE combined with other treatments [3, 12].

An electric field threshold (EFT) model was used to define the difference between the live and dead zones. To the IRE treatment on a particular tumor, the EFT was determined by comparing the dead zone between the computer simulation and the in vivo/in vitro experiment. The value of EFT was defined as the electric field strength that encloses the same or approximate dimension of the dead zone with the experiment. Therefore, in the EFT model, the zones with the electric field strengths higher than a predefined threshold were considered to be effectively ablated or ‘dead’, otherwise they were considered to be ‘live’.

Nickfarjam *et al*. [10] used an EFT of 900 V/cm to differentiate the live and dead zones by comparing the in vivo measurements and the simulation of IRE for a subcutaneous tumor. Similarly, Jiang et al. [13] used an electric field of 600 V/cm as the value of EFT for prostate cancer, based on the fact that nearly 60% of the tumor area in in vitro experiments is correspondent to the estimated injury zone with a radius of 3 mm electric strength circle. Garcia *et al*. [14] chose to use an electric field of 500 V/cm as the EFT of human brain to calculate the volumes of the dead zones with different pulse strengths. Using a rabbit liver model, Miklavčič *et al*. [15] confirmed that the value of EFT varied from 594 V/cm to 680 V/cm under different electric strengths (860–1360 V) while other pulse-setting parameters were set as constants. According to these previous studies, it is clear that the value of EFT is relevant to not only the tissue type but also the IRE-setting parameters. Therefore, the value of EFT for every IRE protocol applied in a computer simulation must be determined in advance using the corresponding in vitro/in vivo experiments, which will increase the cost and difficulty in the application of computer simulation of IRE treatments. The second shortcoming with the EFT model is that it cannot show all the zones in IRE due to its inability to define the transition zone between the live and dead zones, while the transition zone is a key factor to local recurrence after IRE treatments. Indeed, the EFT model uses a ‘black-or-white’ judgment criteria or strategy [16], which inherently limits its validity in representing the outcome of IRE.

To solve the problems with the EFT model, Golberg and Rubinsky [17] proposed a statistical model describing the relationship between the pulse number and cell death for the evaluation of tissue ablation in IRE, and showed that the statistical model can predict the tissue destruction more accurately, compared with the EFT model. However, the data of the cell death they used was adopted from the experiments of prostate cancers treated with ECT instead of IRE, so results achieved by their work cannot be generalized to the IRE treatment. Thus, a statistical model describing the relationship between the tumor cell death and the IRE pulse-setting parameters merits further study. Furthermore, the pulse length is another key factor that should be taken into consideration in the statistical model as well.

In the study reported in the present paper, a statistical model was developed, which represents the relationship between the pulse-setting parameters (i.e., the pulse length and the number of pulses) and the cell death with the IRE treatment data on the cervical cancer cell line (i.e., HeLa cell) and to show the superiority of the statistical model to the EFT model by comparing the results obtained from both models.

## Materials and methods

### Tumor cell culture

Human cervical cancer (HeLa) cell line (purchased from Kingmorn life science, Shanghai, China) was cultured as adherent monolayer in Dulbecco’s modified Eagle’s medium (DMEM)/High Glucose supplemented with 10% fetal bovine serum and 1% penicillin/streptomycin (PS). The cells were grown in culture at 37℃ in 5% CO2 in a humidified incubator. Following 3-mL phosphate buffered saline (PBS) washes, cells were harvested for passaging (split 1:2 or 1:4) or experiments at a confluence of >80% in 100-mm Petri dishes using a 0.25% GIBCO® trypsin-EDTA solution.

### IRE experimental set-up and protocols

After the harvest as above, cells were collected from suspensions by centrifuging them at 1500 r/min for 3 min and supernatant was removed. Cells were then re-suspended in the fresh culture medium as described above to a cell density of 1–4×10^6^ cells/mL. The number and density of cells were monitored by an automated cell counter (Countstar® BioTech, Shanghai Ruiyu Biotech. Co., Ltd., Shanghai, China) using Trypan blue staining technique. The cell sizes were also measured by the cell counter and the cell microscopic images were captured and processed by its built-in software.

**Fig 1:**
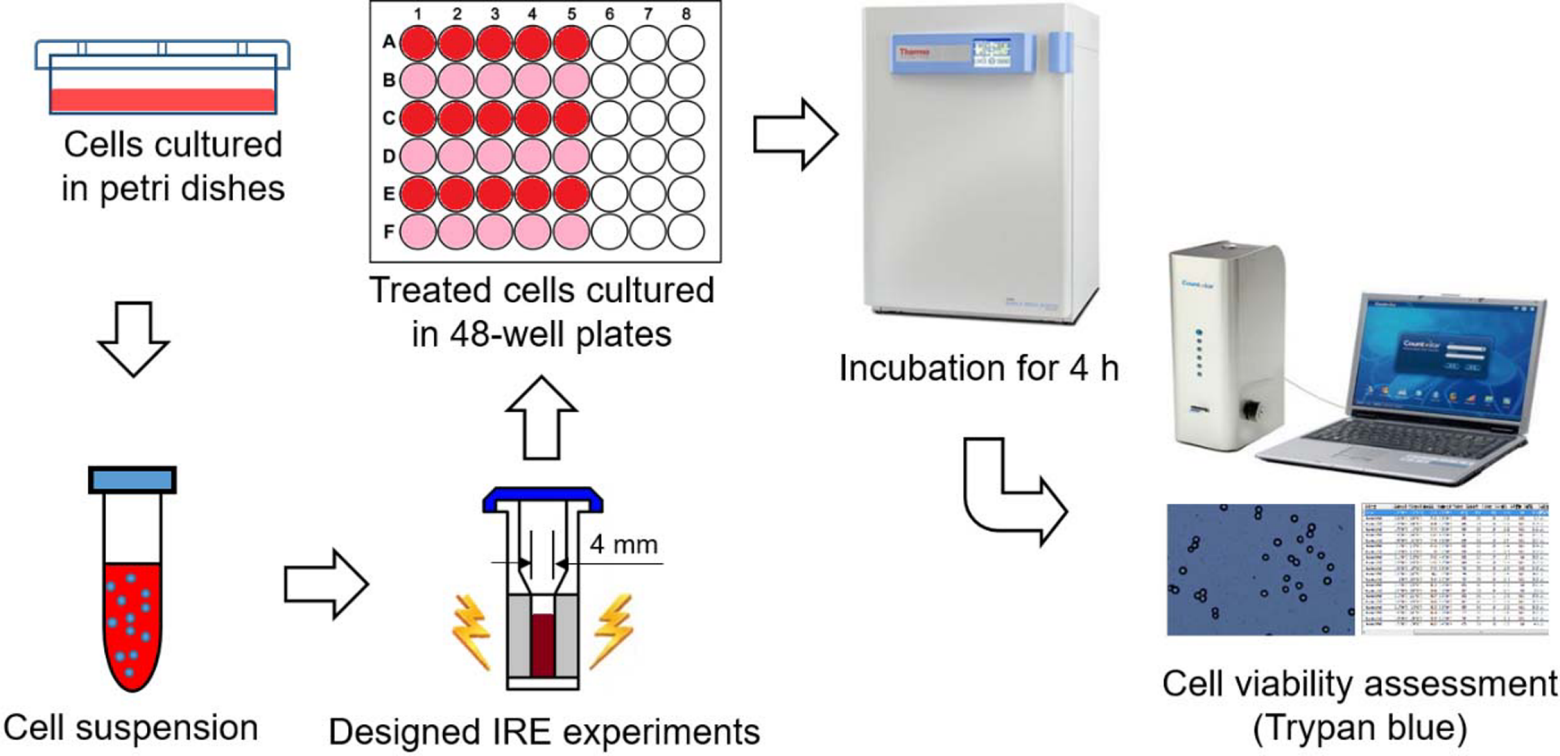
Schematic diagram of IRE experimental set-up and process.

Fig 1 shows the experimental set-up and process of IRE treatment and cell viability assessment. For each IRE protocol, 400 μL of prepared cell suspension was pipetted to an electroporation cuvette (Cuvettes PlusTM 640, Harvard Apparatus, Holliston, MA, USA) with a gap of 4 mm, which was placed in an electric shock chamber (BTX Safety Stand 630B, Harvard Apparatus, Holliston, MA, USA) connected to an IRE pulse generator (BTX ECM 830, Harvard Apparatus, Holliston, MA, USA) for the application of IRE pulses. For the control group, the same amount of prepared cell suspension was pipetted to the cuvette, which was also placed in the chamber for 1–60 seconds without the application of electric pulses. After the IRE treatment, the cell suspension was removed to a well of 48-well plate using pipettes for a 4-h incubation as mentioned above prior to the cell viability assessment (discussed in the section of cell viability assessment). IRE was applied at a room temperature of 20 ± 1°C. Cuvettes were washed by 75% alcohol and then rinsed by deionized water for the next IRE application.

The pulse length and the number of pulses were deliberately selected to be investigated for their statistical relationships with the cell death. Electric pulses were administrated with each of four pulse lengths (25, 50, 75, and 100 μs) at the frequency of 1 Hz, which is close to the clinical-used ECG-synchronized frequency [18]. For each pulse length, one, ten, thirty, and sixty pulses were delivered, respectively. For each ten-, thirty-, and sixty-pulse case, the pulse strengths of 200, 400, 600, and 800 V were applied, which correspond to the electric fields of 500, 1000, 1500, and 2000 V/cm, respectively. To make the cell death more obvious, an additional pulse strength of 1800 V (corresponding to the electric field of 4500 V/cm) was also applied in the one-pulse case. For each protocol, the same IRE test was performed in quintuplicate (N = 5). Therefore, a total number of 340 IRE tests were conducted to obtain the cell death data for the statistical modeling in the study.

### Cell viability measurement

In the study, cell viability for each IRE experiment (including the control group) was evaluated by the Trypan blue staining technique using the automated cell counter as mentioned above. The blue dye can enter the cell to stain the nucleus blue when the cell membrane is incomplete. However, there are two issues that could affect the accuracy of cell viability measurement, such as the morphology of treated cells that could deceive the cell counter leading to an inaccurate counting and the time of introducing blue dye to the treated cells. As mentioned before, pore formation does not necessarily result in cell death due to the existence of reversible electroporation. So for the classic membrane integrity dye assay, determining the time that the dye being introduced is crucial to the accurate measurement of cell viability. To overcome these two issues, a focused study was conducted to determine the optimal time at which the blue dye should be introduced to most accurately determine cell death. In the present study, the blue dye was introduced to the treated cell suspension at 0-, 0.5-, 1-, 2-, 3-, or 4-h incubation, respectively, after the IRE treatment. All the incubation times were investigated using four groups of IRE pulse-setting parameters: Test 1: pulse strength of 1000 V and 30 pulses, Test 2: pulse strength of 1000 V and 60 pulses, Test 3: pulse strength of 1250 V and 30 pulses, and Test 3: pulse strength of 1250 V and 60 pulses. The other pulse-setting parameters were set as: the pulse frequency of 1 Hz and the pulse length of 100μs. Each experiment in the focused study was also performed in quintuplicate and the result was given as mean ± standard deviation. The one-way ANOVA was employed to assess the statistically significant difference of the results using Minitab 17 (Minitab Inc., State College, PA, USA). Results were considered as statistically significant when P <0.05.

For all tests, the cell viabilities measured at 0-, 1-, and 2-h incubation after IRE treatment by Trypan blue were significantly different (Test 1: P <0.003, P <0.001, and P <0.001, respectively; Test 2: P <0.001, P <0.001, and P <0.002, respectively; Test 3: P <0.001, P = 0.001, and P = 0.001, respectively; and Test 4: P <0.001, P = 0.167, and P <0.01, respectively) as shown in S1 Fig. However, there is no significant difference in cell viability for all tests after 2-h incubation or longer. S2 Fig shows the change of cell morphology after 0-, 1-, 2-, 3-, and 4-h incubation for Test 2. It can be found that with the increase in the incubation time, the cells treated with IRE were clearly distinguishable from those with RE. The treated cells after the 4-h incubation were distinct from the transition phase (no transition cells found). Besides, the cell viability measured after 4-h incubation was quite similar with that achieved by the previous studies under the same pulse-setting conditions for different cell types (HeLa cells: 9.631% ± 2.81% vs. PC3 cells: 27% ± 9% [19] and HeLa cells: 75.93% ± 4.09% vs. DU 145 prostate cancer cells: 80% ± 6% [20]). Therefore, the viability of treated cells was measured by the Trypan blue staining technique after a 4-h incubation after the IRE treatment in the present study.

### Statistical model of cell death

Many studies on electroporation to treat the cells in the food industry showed that the death of a heterogeneous population of cells was a statistical event [21–23]. There are many mathematical models proposed in literature to describe the survival of cells due to electroporation, like the first-order kinetics [24], the Peleg-Fermi [25], the Weibull [26], the logistic [27], the adapted Gompertz [28], and the Geeraerd [29] models. Dermol *et al*. conducted a comparison study of several commonly-used statistical models, concluding that the adapted Gompertz and the Geeraerd models are suitable for describing the cell survival as a function of treatment time, while the Peleg-Fermi, the adapted Gompertz, and the logistic models can all be used as function of electric field for describing cell death due to electroporation.

Thus, considering the number of pulses and the pulse length that are two investigated pulse-setting parameters in the study, the Peleg-Fermi model [25] was used to describe the cell death on the number of pulses and electric field strength for a given pulse length in this study, which was given as:

$$\text{S}=\frac{100}{1+{\text{e}}^{\left(\frac{\text{E}-{\text{E}}_{\text{c}}}{\text{A}}\right)}}$$ (1)

where, S is the ratio of live cells after the IRE treatment; E (V/cm) is the electric field, E_c_ is the electric field at which 50% of a population of cells are dead; A (V/cm) is a parameter indicating the steepness of the survival curve at the electric field of E_c_. It is worth noting that the exponential function can amplify error. According to the previous studies [25], both E_c_(n,t) and A(n,t) as exponential functions of the number of pulses and the pulse duration were also determined by the pulse and the cell types. In the study, the pulse type and the cell type were set as squared wave and HeLa cell, respectively. Both E_c_(n,t) and A(n,t) were evaluated at a particular pulse length (t becomes a constant):

$${\text{E}}_{\text{c}}\left(\text{n}\right)={\text{E}}_{\text{c}1}{\text{e}}^{-{\text{k}}_{1}\text{n}}+{\text{E}}_{\text{c}2}{\text{e}}^{-{\text{k}}_{2}\text{n}}$$ (2)

$$\text{A}\left(\text{n}\right)={\text{A}}_{1}{\text{e}}^{-{\text{k}}_{3}\text{n}}+{\text{A}}_{2}{\text{e}}^{-{\text{k}}_{4}\text{n}}$$ (3)

where E_c1_ (V/cm) and E_c2_ (V/cm) are the electric field at which 50% of a population of cells are dead; A_1_ (V/cm) and A_2_ (V/cm) are the steepnesses of the survival curve at the electric fields of E_c1_ and E_c2_, respectively; k_1_, k_2_, k_3_ and k_4_ are dimensionless constants; n is the number of pulses.

Or they are evaluated at a particular number of pulses (n becomes a constant):

$${\text{E}}_{\text{c}}\left(\text{t}\right)={\text{E}}_{\text{c}3}{\text{e}}^{-{\text{k}}_{5}\text{t}}+{\text{E}}_{\text{c}4}{\text{e}}^{-{\text{k}}_{6}\text{t}}$$ (4)

$$\text{A}\left(\text{t}\right)={\text{A}}_{3}{\text{e}}^{-{\text{k}}_{7}\text{t}}+{\text{A}}_{4}{\text{e}}^{-{\text{k}}_{8}\text{t}}$$ (5)

where E_c3_ (V/cm) and E_c4_ (V/cm) are the electric field at which 50% of a population of cells are dead; A_3_ (V/cm) and A_4_ (V/cm) are the steepnesses of the survival curve at the electric fields of E_c3_ and E_c4_, respectively; k_5_, k_6_, k_7_ and k_8_ are dimensionless constants; and t is the pulse length.

Therefore, the Peleg-Fermi model, Eq (1) can become to Eq (6) or Eq (7) by substituting Eqs (2) and (3) and Eqs (4) and (5), respectively

$$\text{S}\left(\text{E},\text{n}\right)=\frac{100}{1+{\text{e}}^{\left(\frac{\text{E}-{\text{E}}_{\text{c}1}{\text{e}}^{-{\text{k}}_{1}\text{n}}-{\text{E}}_{\text{c}2}{\text{e}}^{-{\text{k}}_{2}\text{n}}}{{\text{A}}_{1}{\text{e}}^{-{\text{k}}_{3}\text{n}}+{\text{A}}_{2}{\text{e}}^{-{\text{k}}_{4}\text{n}}}\right)}}$$ (6)

$$\text{S}\left(\text{E},\text{t}\right)=\frac{100}{1+{\text{e}}^{\left(\frac{\text{E}-{\text{E}}_{\text{c}3}{\text{e}}^{-{\text{k}}_{5}\text{t}}-{\text{E}}_{\text{c}4}{\text{e}}^{-{\text{k}}_{6}\text{t}}}{{\text{A}}_{3}{\text{e}}^{-{\text{k}}_{7}\text{t}}+{\text{A}}_{4}{\text{e}}^{-{\text{k}}_{8}\text{t}}}\right)}}$$ (7)

In present study, Eqs (6) and (7) were applied to build the statistical relationship between the cell death and the number of pulses and the relationship between the cell death and the pulse length, respectively.

### Numerical modeling of IRE and EFT model

The electric field distribution during IRE was determined by solving the Laplace equation [14]:

−∇(σ(E)∇φ)=0 (8)

where φ is the electrical potential; E is the applied electric field; and σ(E) is the electric-field-dependent electrical conductivity of the tissue. The relationship between the electric field strength and the electrical conductivity for cervical cancer used in this study was described by using the asymmetrical sigmoid Gompertz curve function [30]:

$$\sigma \left(\text{E}\right)=\sigma_{0}+\left(\sigma_{\text{max}}-\sigma_{0}\right){\text{e}}^{-{\text{ae}}^{\left(-\text{bE}\right)}}$$ (9)

where σ_0_ and σ_max_ are the minimum (no electroporation) and maximum (fully saturated) electrical conductivities within a single pulse respectively; a and b that vary with pulse length (t) are unitless coefficients for the displacement and growth rate of the curve, respectively.

$$\text{a}=-5\times 10^{-6}{\text{t}}^{2}+0.004\text{t}+2.803$$ (10)

$$\text{b}=-7\times 10^{-9}{\text{t}}^{2}+5\times 10^{-6}\text{t}+0.002$$ (11)

**Fig 2:**
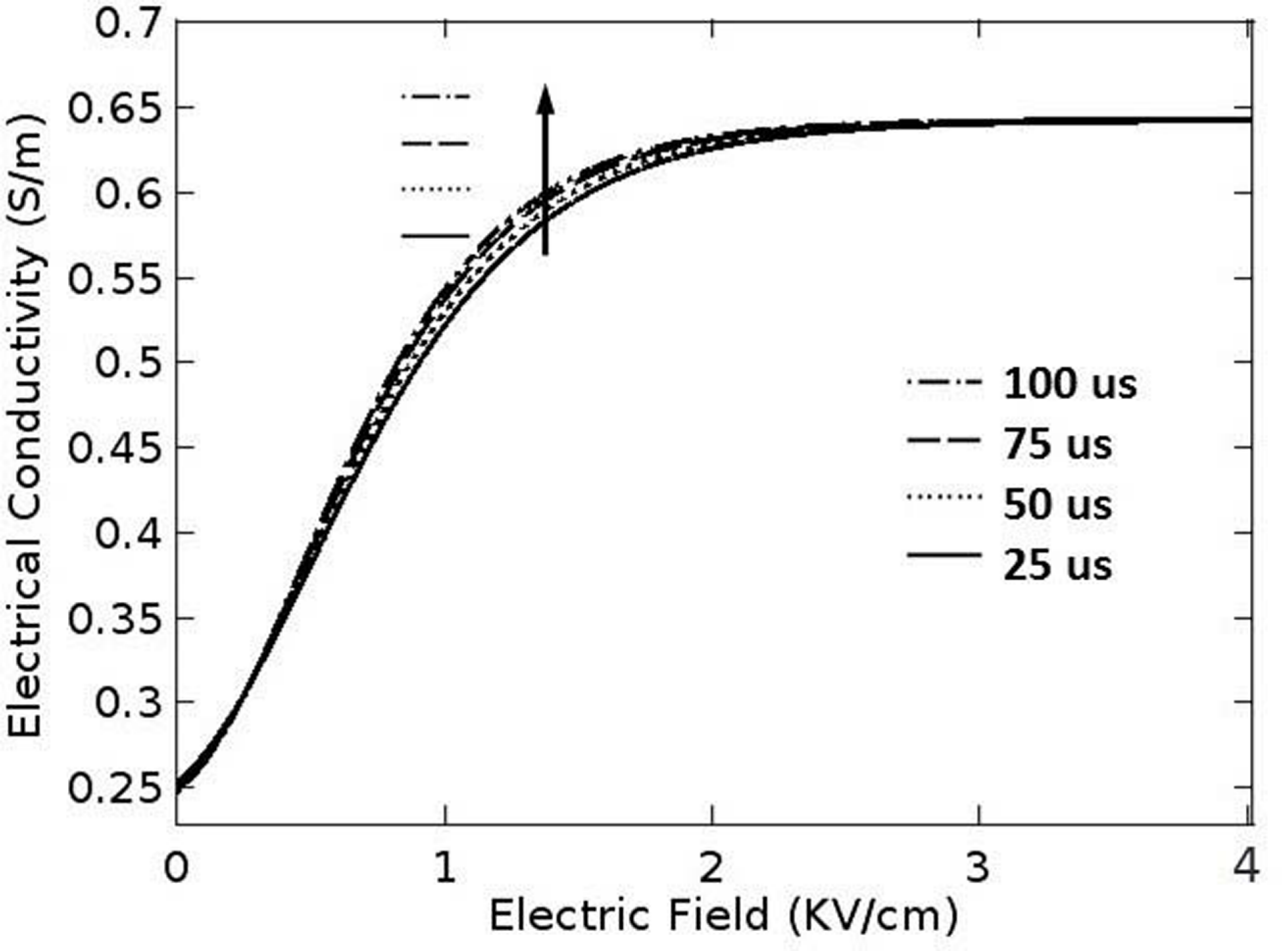
Electric-field-dependent electrical conductivities of cervical cancer for four pulse lengths.

For four pulse lengths used in the study, the values of σ_0_, σ_max_, a, and b were tabulated in Table 1. The electric conductivity of healthy cervical tissue is 0.2033 S/m at 1 Hz [31]. In the present study, the electric conductivity of the cervical cancer was determined by increasing the electric conductivity of healthy cervical tissue by the factor of 1.13 [32]. Due to the lack of data, σ_max_ was determined by increasing σ_0_ by the factor of 1.8 [33]. Fig 2 shows the electric-field-dependent electrical conductivities of cervical cancer for the four pulses lengths used in the study.

**Table 1:** Values of σ_0_, σ_max_, a, and b used in the study.

Pulse Length (μs)	σ_0_ (S/m)	σ_max_ (S/m)	a	b
25	0.22973	0.64324	2.89988	0.00212
50			2.99050	0.00223
75			3.07487	0.00234
100			3.15300	0.00243

In the present study, a two-dimensional finite element model with a length and width of 100 mm and 80 mm, respectively was used to solve the electric field distribution after the IRE treatment. To avoid the boundary effect, the dimension of the two-dimensional model was determined by a sensitive study. The model was solved in the COMSOL Multiphysics 5.2a (COMSOL, Inc., Burlington, MA, USA) software to get the electric field distribution. The material of this model was set as the cervical cancer tissue. The electric pulses were applied using two 1-mm electrodes with a center-to-center distance of 10 mm acting as the anode and the cathode, respectively.

To solve Eq (8), the electrical boundaries of the electrodes were given as:

$$\varphi =\{\begin{array}{c} V_{0}\;\text{for anode}\\ 0\;\text{for cathode} \end{array}$$ (12)

Electrical boundary of the outer surface of tissue was treated as electrically insulated:

$$\frac{\partial \varphi }{\partial n}=0$$ (13)

**Fig 3:**
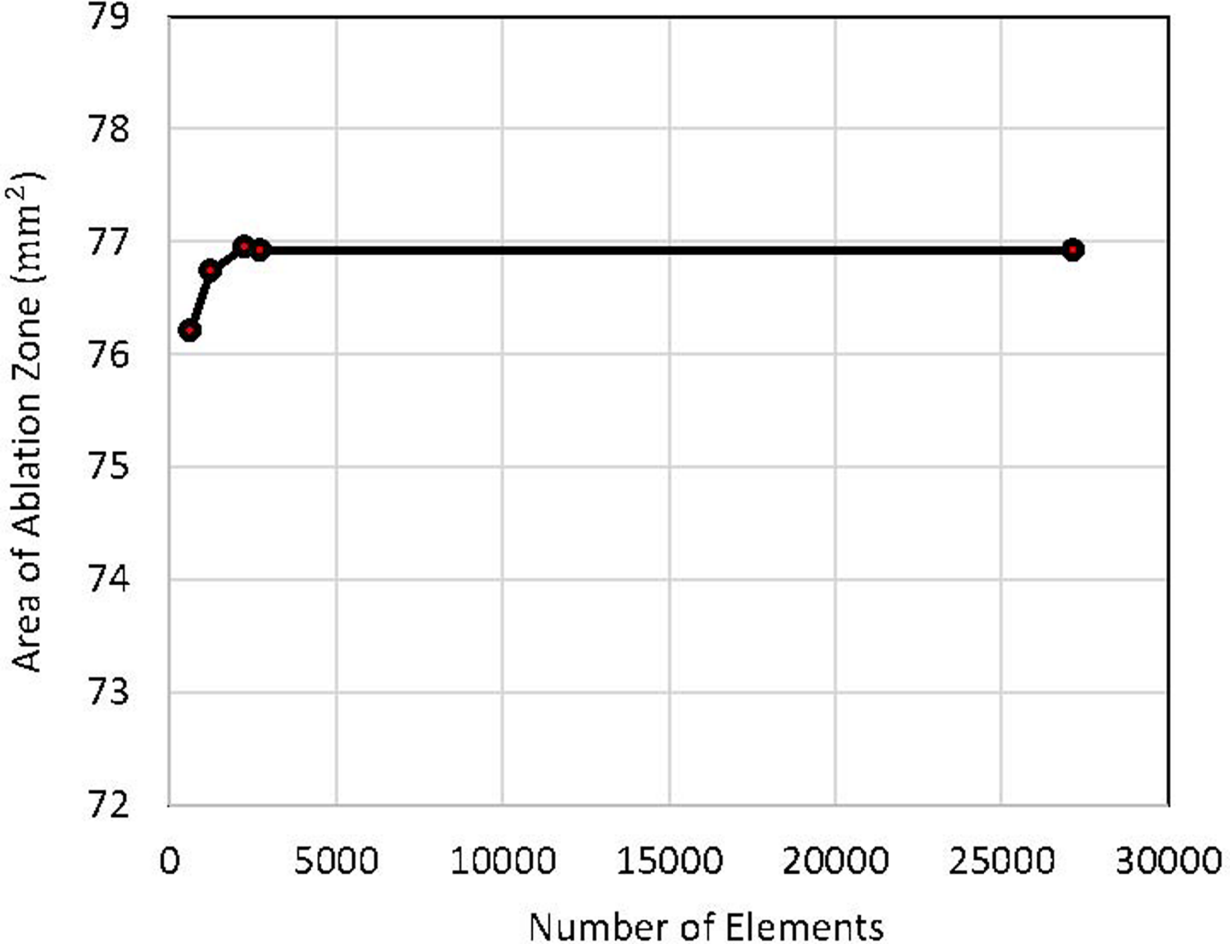
Results of mesh convergence test.

The heat damage was not taken into consideration in the study, though it can be impressive if very high electric pulses are applied, which was not the case in the present study. A convergence test of the finite element model on the number of elements was performed to obtain a stable mesh, which means that the electric field distribution is independent of the number of elements in the study. Eight electric pulses with the pulse strength of 1000 V and the pulse length of 100 μs were applied at the frequency of 1 Hz for the convergence test. Considering the goal of this study was to have an accurate model to calculate the tissue death in IRE treatment, we used the size of ablation zone (EFT model) as an objective item to evaluate the mesh. The value of EFT that leads to death of tissue was set as 600 V/cm. Specifically, the mesh was continually refined until the change of the ablation zone in the model is within 0.1% between the two consecutive meshes. The results of mesh convergence test were given in Fig 3. When the number of elements exceeds 2710, the change in the area of ablation zone was less than 0.1%. Thus, a mesh with 2710 elements was used in the model.

## Results

### Statistical model of cell death

**Fig 4:**
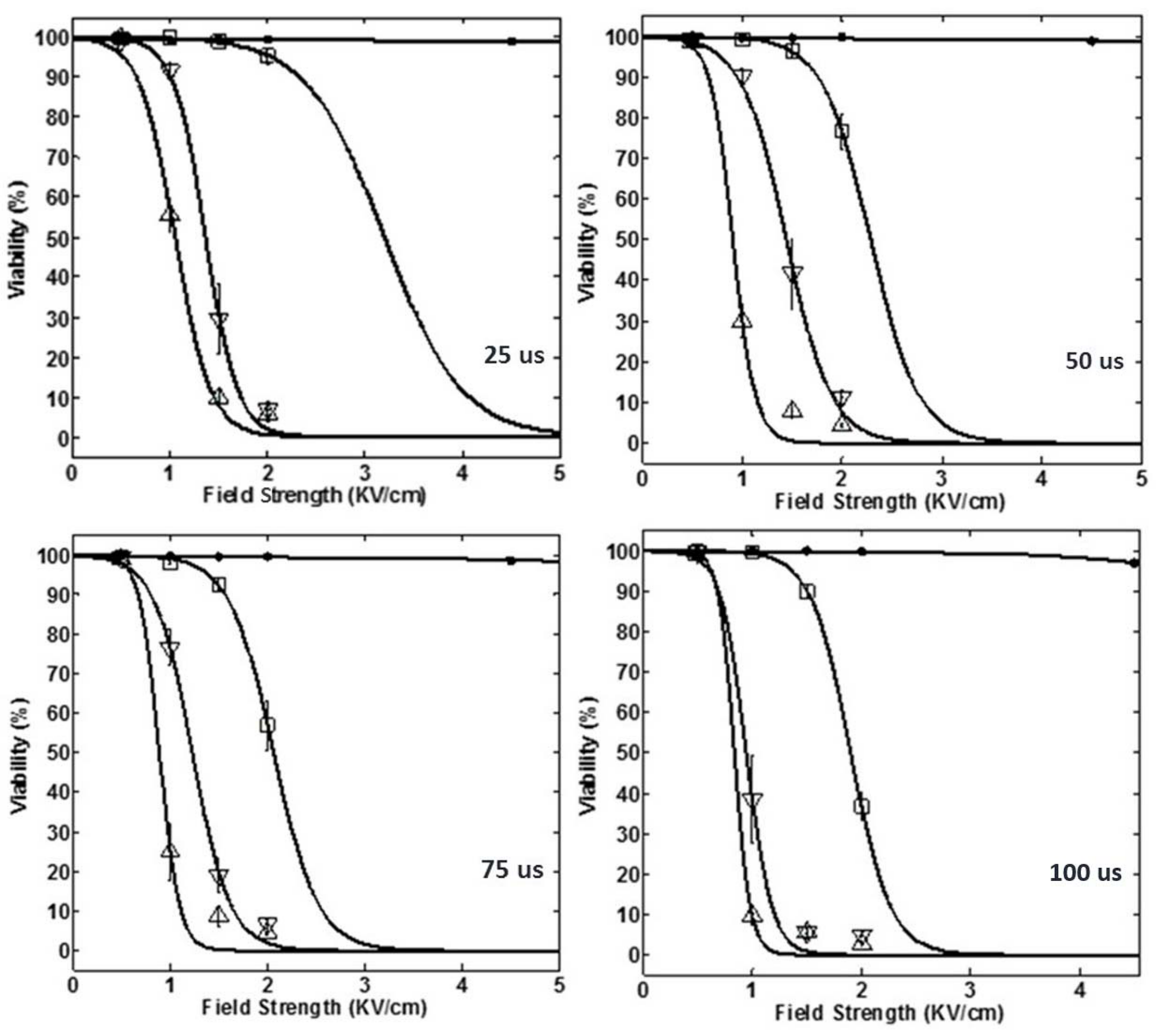
Cell viability dependence on the field strength and each of the four used number of pulses (‘●’, ‘□’, ‘▽’ and ‘△’ represent 1, 10, 30, and 60 pulses, respectively) at different pulse lengths: a) 25 μs, b) 50 μs, c) 75 μs, and d) 100 μs, respectively.

**Fig 5:**
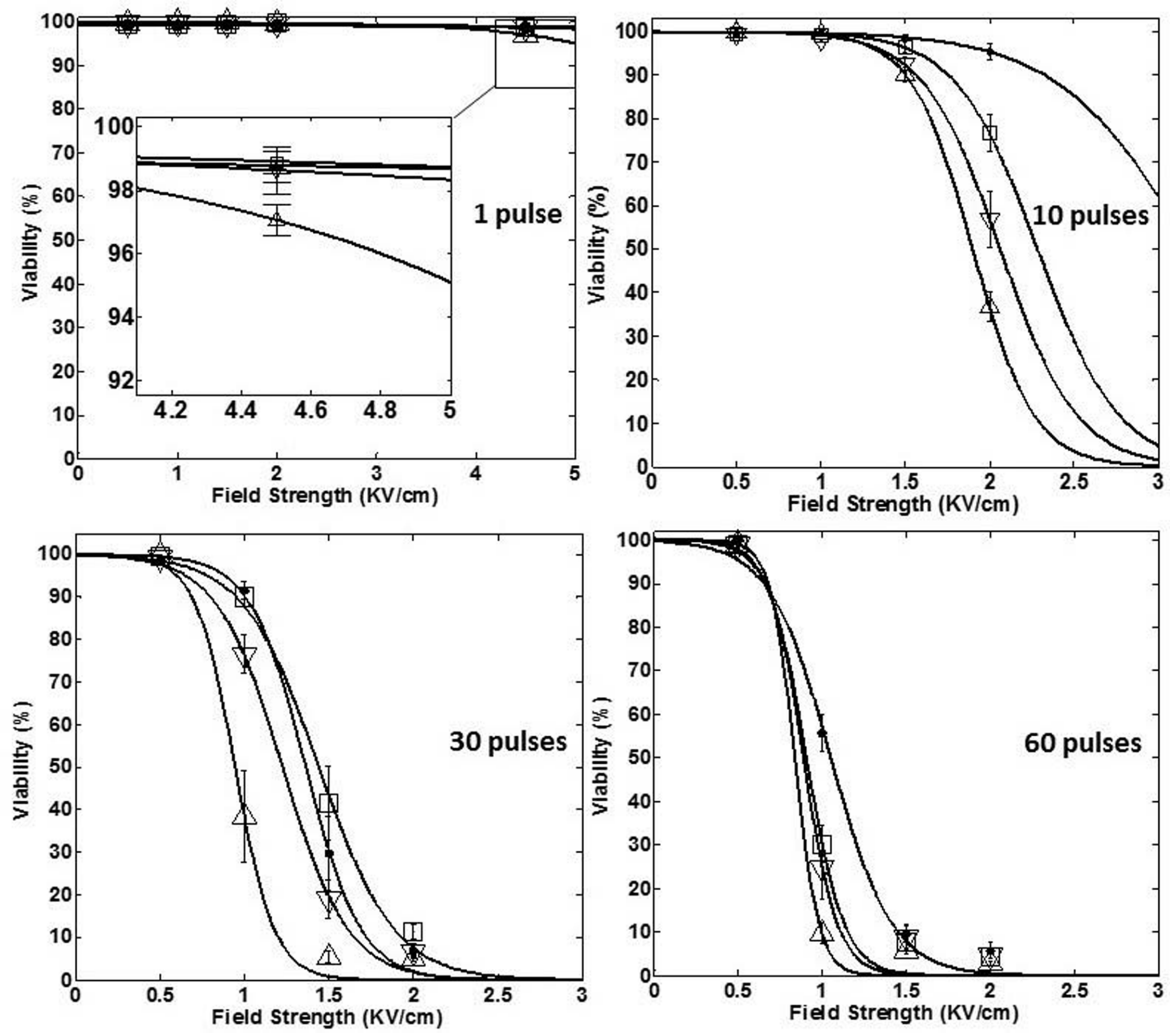
Cell viability dependence on the field strength and each of the four used pulse lengths (‘●’, ‘□’, ‘▽’ and ‘△’ represent 25, 50, 75, and 100 μs, respectively) for different number of pulses: a) 1, b) 10, c) 30, and d) 60, respectively.

The viability of HeLa cells treated with different IRE protocols was calculated by comparing with the viability of its control group and given in S1 Table. Fig 4 shows the cell viability dependence on the field strength and the pulse length for each of four-used number of pulses, like 1, 10, 30, and 60 pulses, as shown in Fig 4A, 4B, 4C and 4D, respectively. Fig 5 shows the cell viability dependence on the field strength and the number of pulses for each of four-used pulse length, like 25, 50, 75, and 100 μs, as shown in Fig 5A, 5B, 5C and 5D. The curves shown in Figs 4 and 5 were fitted by the Peleg-Fermi model using Eq (1). It is obvious that the steepness of curves increased with an increase in the pulse strength or the number of pulses, respectively which means the faster ablation effect of IRE.

**Fig 6:**
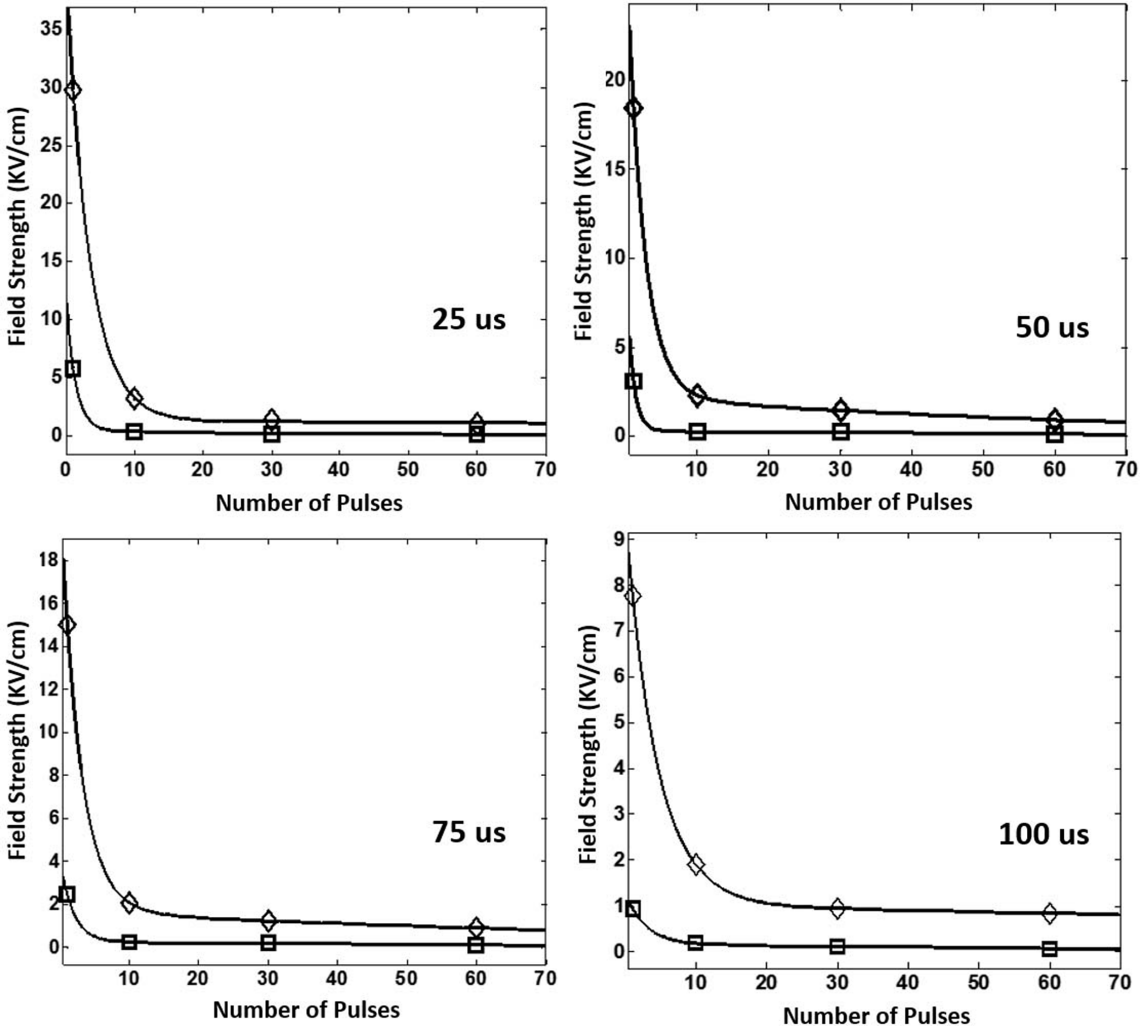
Dependence of E_c_ (‘◇’) and A (‘□’) on the number of pulses.

**Fig 7:**
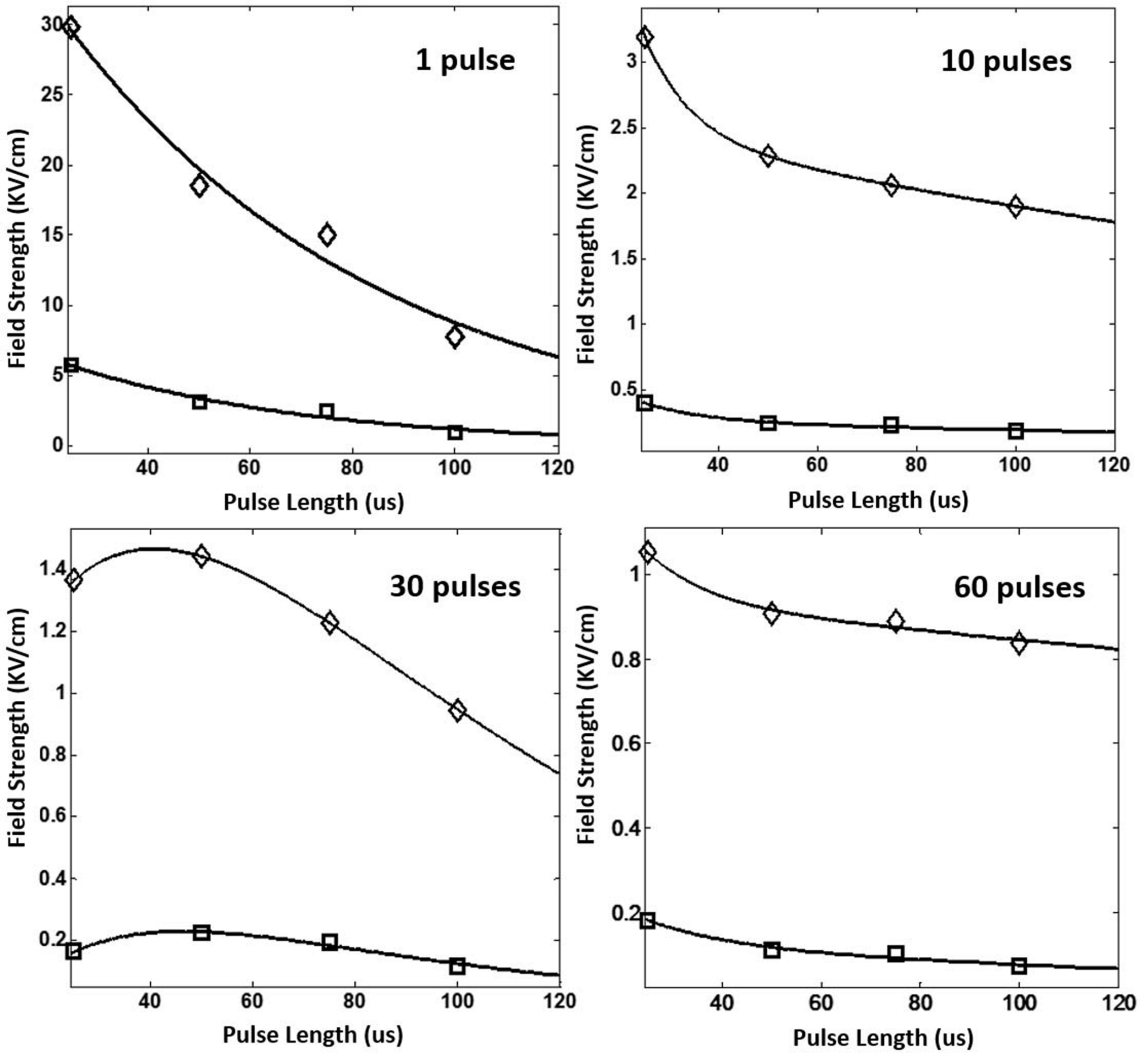
Dependence of E_c_ (‘◇’) and A (‘□’) on the pulse length.

Based on the data, the curve fitted parameters E_c_ and A as functions of the number of pulses (n) and the pulse length (t) were estimated and as shown in Fig 6 (E_c_(n) and A(n)) and Fig 7(E_c_(t) and A(t)), respectively. The values of fitting parameters for E_c_(n) and A(n) as well as E_c_(t) and A(t) were tabulated in Tables 2 and 3, respectively. It is noted that, with two exponential components, a very good fitness (see the value of R^2^ in Tables 2 and 3) was able to be achieved for both E_c_ and A (i.e., Eqs (2)–(5)) under each of those pulse length and pulse number conditions. It merits mentioning that the good finesses of E_c_ and A are critical for the accuracy of cell death in the statistical model of IRE.

**Table 2:** Curve fitting results for E_c_(n) and A(n) at different pulse lengths.

Pulse length (μs)	E_c_(n)					A(n)				
	E_c1_ (V/cm)	K_1_	E_c2_ (V/cm)	K_2_	R^2^	A_1_ (V/cm)	K_3_	A_2_ (V/cm)	K_4_	R^2^
25	38.310	-0.2953	1.280	-0.001815	1.00	0.4442	-0.02095	11.0300	-0.73010	0.9996
50	24.910	-0.4257	2.250	-0.015030	1.00	0.2916	-0.01354	8.4900	-1.10700	0.9903
75	19.040	-0.3533	1.678	-0.010530	1.00	3.8130	-0.54730	0.2533	-0.01314	0.9998
100	8.428	-0.2237	1.038	-0.003552	1.00	1.0790	-0.33910	0.1678	-0.01329	0.9999

**Table 3:** Curve fitting results for E_c_(t) and A(t) at different numbers of pulses.

Pulse #	E_c_(t)					A(t)				
	E_c3_ (V/cm)	K_5_	E_c4_ (V/cm)	K_6_	R^2^	A_3_ (V/cm)	K_7_	A_4_ (V/cm)	K_8_	R^2^
1	44.3000	-0.01620	0	0	0.9760	9.6030	-0.02092	0	0	0.9709
10	11.7700	-0.10880	2.6520	-0.00323	1.0000	1.0010	-0.07780	0.2776	-0.003821	0.9847
30	12.9700	-0.01840	-12.4900	-0.02421	1.0000	3.1940	-0.02519	-3.4880	-0.032750	0.9681
60	0.9476	-0.08218	0.9599	-0.00128	1.0000	0.2557	-0.05917	0.1441	-0.006390	0.9800

It is worth mentioning that the death of treated cells was not getting to zero even though the pulses with high pulse strength were used in the in vitro experiment, which is, we believe, due to the measurement accuracy of the cell counter machine. So in the present study, the cell area with less than 1% viability was taken as dead. Eq (14) was used to calculate the area of ablation zone for the proposed statistical model in the study.

S=∬Viability(x,y)≤1% dxdy (14)

### Case study

**Fig 8:**
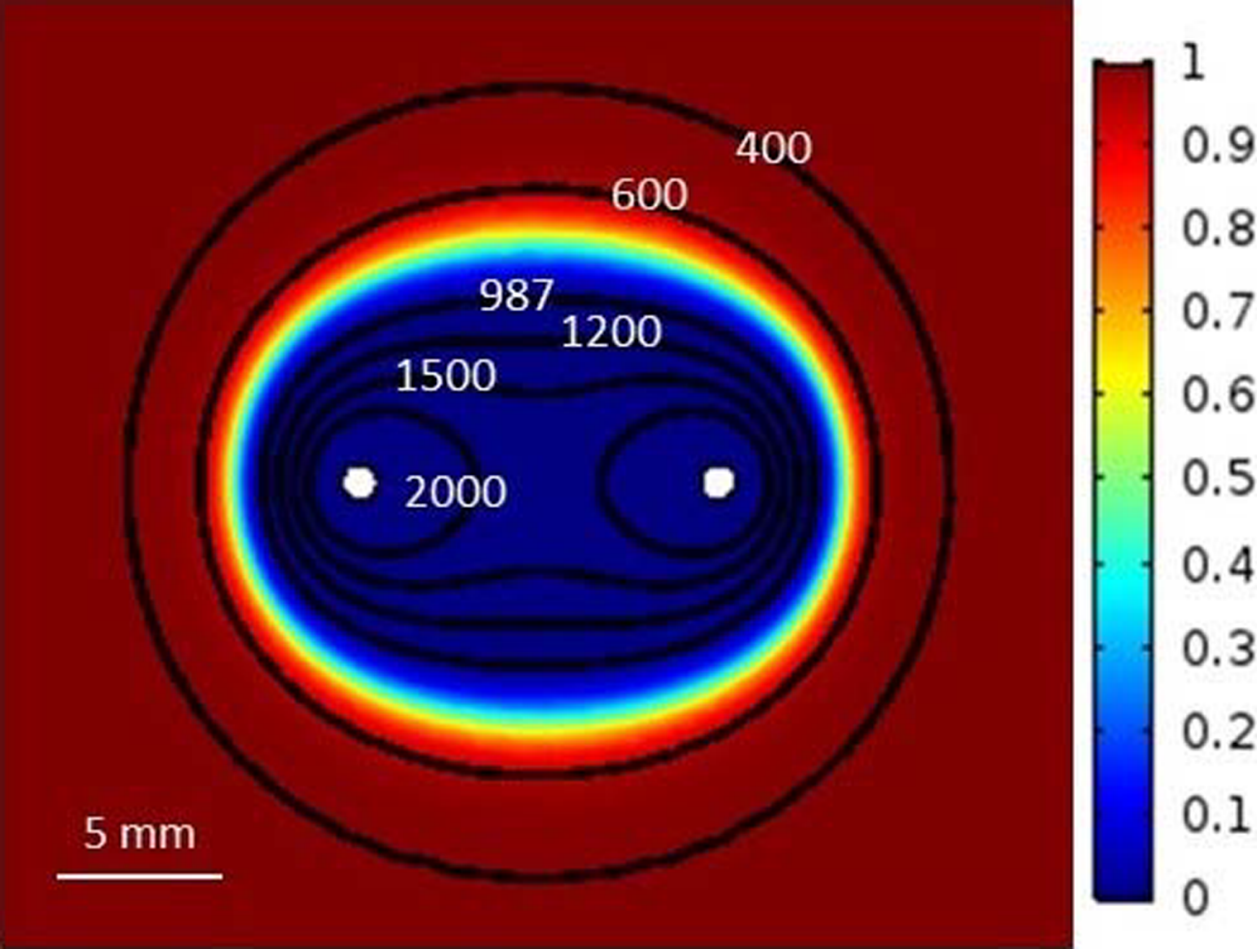
Comparison of ablation zones between the statistical model and the EFT model (electric field strength in V/cm).

Two case studies were also performed to investigate the accuracy of statistical model in the expression of ablation zone and its applicability for different pulse-setting parameters. Fig 8 shows the comparison of ablation zones between the statistical model and EFT model when 90 pulses with the pulse strength of 2500 V and the pulse length of 100 **μ**s were applied at the frequency of 1 Hz. According to Eq (7), the ablation zone of statistical model was covered by the corresponding electric field of 987 V/cm in the EFT model. It is easy to find that there was a transition zone between the ablation zone and intact zone in the statistical model, which was not able to be found in the EFT model, as shown in Fig 8. Similarly, the values of EFT for the cervical cancer under different pulse-setting parameters were also calculated and tabulated in Table 4. As shown in Table 4, one could find that the value of EFT changes with the IRE-setting parameters, which proves the above-mentioned statement on the disadvantages of EFT model.

**Table 4:** Values of EFT for the cervical cancer under different pulse setting parameters.

Pulse strength (V)	Number of pulses	Pulse length (μs)	Pulse frequency (Hz)	Ablation zone (mm^2^)	Value of EFT (V/cm)
2000	90	100	1	95.115	988
2500	90	100	1	127.780	987
2500	60	100	1	97.978	1186
2500	30	100	1	69.379	1461
2500	90	75	1	125.190	1007
2500	90	50	1	130.380	978
2500	90	25	1	76.248	1397
3000	90	100	1	158.36	987

**Fig 9:**
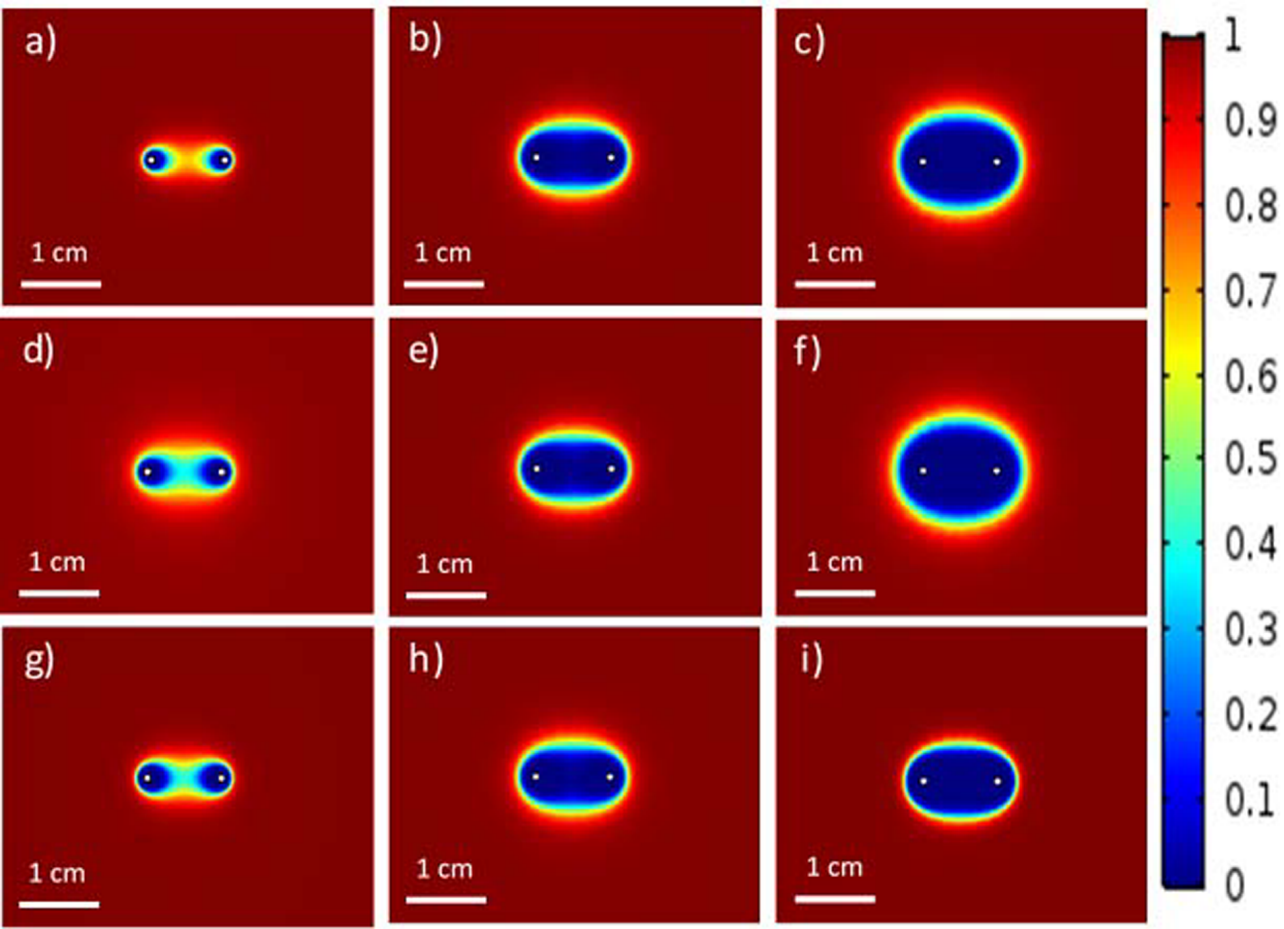
Viability plots for IRE in cervical tumor tissues for different IRE settings regarding to the number of pulses, the pulse strength, and the pulse length: a) 60, 1000 V, and 50 μs, b) 60, 2000 V, and 50 μs, c) 60, 3000 V, and 50 μs, d) 30, 2000 V, and, 50 μs, e) 60, 2000 V, and, 50 μs, f) 90, 2000 V, and 50 μs, g) 60, 2000 V, and 10 μs, h) 60, 2000 V, and 50 μs, and i) 60, 2000 V, and 100 μs, respectively.

Fig 9 shows the changes of ablation zone with the changes in the pulse-setting parameters using the proposed statistical model. A significant increase in the ablation zone was found when there was an increase in the pulse strength, the number of pulses, or the pulse length. Fig 9A, 9B and 9C were the ablation zones when the pulse strength was set as to 1000, 2000, and 3000 V, respectively using 60 pulses with the pulse length of 50 μs. Fig 9D, 9E and 9F were the ablation zones when the number of pulses was set as to 30, 60, and 90, respectively using the pulse strength of 2000 V with the pulse length of 50 μs. Similarly, the ablation zone also increased with the increase in the pulse strength, as shown in Fig 9G, 9H and 9I corresponding to the pulse lengths of 10, 50, and 100 **μ**s, respectively. Therefore, it is safe to conclude that the proposed statistical model has the capability of expressing the change of ablation zone with the change in pulse-setting parameters.

## Discussion

In present study, a statistical relationship between tumor cell death and the IRE-setting parameters (i.e., pulse length, number of pulses, and pulse strength) was built using the data from HeLa cell suspensions treated with various IRE protocols. The Trypan blue staining technique was used to evaluate the cell viability after each IRE treatment. The time of introducing the Trypan blue dye was also investigated, and it can be concluded that the cell viability of HeLa cells treated with IRE is more accurate and robust when a 4-h incubation is performed before the introduction of dye solution. Due to the lack of data, two different cell types treated with the same IRE protocols with this study were checked for the accuracy of cell viability measured in the study.

As shown in Figs 8 and 9, the treatment process of IRE can be more accurately described by the statistical model comparing with the EFT model. Particularly, the statistical model can demonstrate the transition areas of ablation in which the cells are dying or recovering from pores after the removal of electric field. As shown in Fig 9, it is clear that the transition area of ablation becomes larger and larger with the increase in the pulse strength or the pulse width, which is an important piece of information for surgeons and interventional radiologists when determining the pulse-setting parameters clinically. The accurate determination of the transition area is crucial for preventing local recurrence. The proposed statistical model can be used to facilitate treatment planning for surgeons and interventional radiologists. Unlike the EFT model which uses the experiment to determine the electric threshold for cell death, the statistical model can be easily used to determine the electric thresholds for different IRE protocols. A limitation of this study is that the data used for the statistical model is achieved from the HeLa cells treated with IRE protocols. Experiments with in vivo cervical models treated with IRE would be more realistic for the proposed statistical model. The cell viability can be different between the in vitro and the in vivo experiments because the cell microenvironment can affect the result of IRE treatments. Therefore, in vivo experiments with cervical cancers are still necessary in order to construct a more precise IRE treatment plan that can be used clinically. Also, only cervical cancer cells were used in the study. Future studies are warranted to develop statistical models specific to other cancers commonly treated with IRE, such as prostate cancers, breast cancers, liver cancers, pancreas cancers, etc. However, the overall concept should be similar.

## Conclusions

Our conclusions are: (1) the proposed statistical model was able to describe the ablation zone of IRE with cervical cancer cells more accurately compared with the electric field model; (2) the proposed statistical model was able to estimate the value of an electric field threshold for the computer simulation of IRE in the treatment of cervical cancer; and (3) the proposed statistical model was able to express the change of ablation zone with the change in pulse-setting parameters.

## Supporting information

S1 Fig The viabilities of treated HeLa cells at different incubation times in four groups of pulse-setting parameters: a) Test 1, b) Test 2, c) Test 3, and d) Test 4.
(TIF)

S2 Fig The cell morphology of treated HeLa cells in Test 2 at different measure times: a) 0-h incubation, b) 0.5-h incubation, c) 1-h incubation, d) 2-h incubation, e) 3-h incubation, and f) 4-h incubation. Circle: cells under IRE/RE, blank arrow: live cells, and solid arrow: dead cells.
(TIF)

S1 Table Relative cell viability dependence on the pulse strength, the number of pulses, and the electric field strength. (PDF)
