# Supplementary figures and images for: Development of a statistical model for cervical cancer cell death with irreversible electroporation in vitro
Source: PLoS One. 2018 Apr 25;13(4):e0195561. doi: 10.1371/journal.pone.0195561 (PMC5919048; doi:10.1371/journal.pone.0195561)

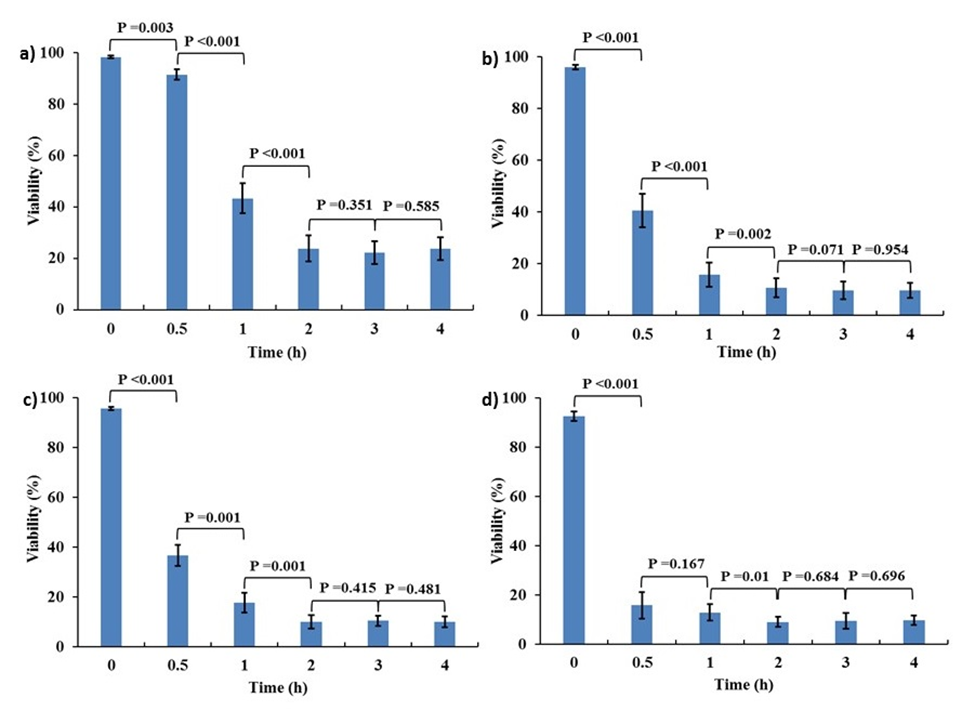

Supplement: S1 Fig — The viabilities of treated HeLa cells at different incubation times in four groups of pulse-setting parameters: a) Test 1, b) Test 2, c) Test 3, and d) Test 4. (TIF) [file pone.0195561.s001.tif]

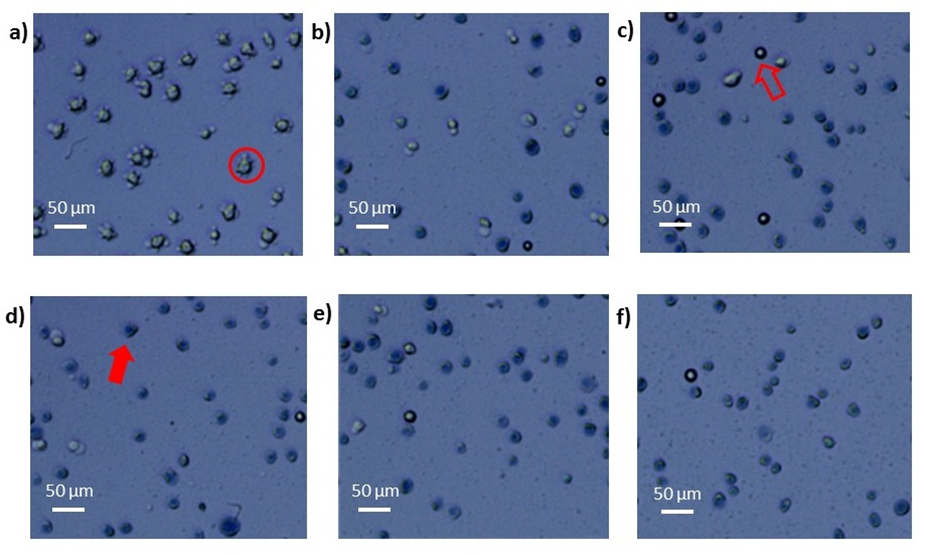

Supplement: S2 Fig — The cell morphology of treated HeLa cells in Test 2 at different measure times: a) 0-h incubation, b) 0.5-h incubation, c) 1-h incubation, d) 2-h incubation, e) 3-h incubation, and f) 4-h incubation. Circle: cells under IRE/RE, blank arrow: live cells, and solid arrow: dead cells. (TIF) [file pone.0195561.s002.tif]
