# Supplementary material for: Development of a statistical model for cervical cancer cell death with irreversible electroporation in vitro
Source: PLoS One. 2018 Apr 25;13(4):e0195561. doi: 10.1371/journal.pone.0195561 (PMC5919048; doi:10.1371/journal.pone.0195561)
Supplement: S1 Table — (PDF) [file pone.0195561.s003.pdf]

S1 Table. Relative cell viability dependence on the pulse strength, the number of pulses, and the electric field strength.

Pulse length (µs)	Number of pulses	Electric field strength (V/cm)	Relative viability of treated cells (%)	Viability of control group (%)
25	1	500	98.358±0.252	98.992±0.127
		1000	98.184±0.107	
		1500	98.318±0.228	
		2000	98.386±0.241	
		4500	97.958±0.445	
	10	500	98.634±0.211	99.110±0.104
		1000	98.848±0.240	
		1500	97.778±0.494	
		2000	94.494±1.989	
	30	500	97.640±0.318	98.734±0.274
		1000	90.340±1.754	
		1500	29.278±8.562	
		2000	6.770±1.669	
	60	500	98.702±0.303	98.754±0.350
		1000	54.974±4.298	
		1500	9.602±1.902	
		2000	5.678±1.727	
50	1	500	98.662±0.149	99.198±0.114
		1000	98.886±0.230	
		1500	98.812±0.150	
		2000	98.930±0.110	
		4500	98.090±0.291	
	10	500	98.442±0.429	99.192±0.071
		1000	98.330±0.259	
		1500	95.658±1.185	
		2000	75.932±4.091	
	30	500	98.608±0.258	99.192±0.071
		1000	89.298±2.179	
		1500	41.278±8.580	
		2000	11.200±1.624	
	60	500	96.434±0.252	97.528±0.169
		1000	29.328±4.145	
		1500	7.592±1.820	
		2000	4.236±0.513	
75	1	500	98.892±0.110	99.198±0.114
		1000	98.902±0.122	
		1500	98.800±0.140	
		2000	98.710±0.250	
		4500	97.850±0.650	
	10	500	98.384±0.116	98.992±0.127
		1000	96.948±0.300	
		1500	91.656±1.460	
		2000	56.350±6.380	
	30	500	98.356±0.359	99.092±0.218
		1000	75.754±4.407	
		1500	18.666±4.389	
		2000	6.590±0.905	
	60	500	98.008±0.322	99.092±0.218
		1000	24.660±7.091	
		1500	8.680±2.488	
		2000	4.556±0.691	

S1 Table. Continued.

Pulse length (μs)	Number of pulses	Electric field strength (V/cm)	Relative viability of treated cells (%)	Viability of control group (%)
100	1	500	98.568±0.385	98.754±0.350
		1000	99.016±0.209	
		1500	98.814±0.235	
		2000	98.744±0.213	
		4500	96.295±0.540	
	10	500	98.924±0.273	98.754±0.350
		1000	98.348±0.143	
		1500	88.928±1.380	
		2000	36.272±3.343	
	30	500	98.666±0.232	98.754±0.350
		1000	37.828±10.722	
		1500	5.254±1.448	
		2000	4.792±0.563	
	60	500	97.87±0.543	98.754±0.350
		1000	9.508±2.466	
		1500	5.544±0.679	
		2000	2.794±0.476	
